# An adult female patient with single atrium and single ventricle undergoing appendectomy: A case report

**DOI:** 10.3389/fcvm.2023.1110269

**Published:** 2023-02-03

**Authors:** Yu Wu, Zenghua Cai, Jianzheng Cheng, Bo Zhang, Guoji Wang, Wei Li, Zaiwang Zhang

**Affiliations:** ^1^Department of Anesthesiology, The 980th Hospital (Bethune International Peace Hospital) of the Joint Logistic Support Force, Shijiazhuang, China; ^2^Department of General Surgery, The 980th Hospital (Bethune International Peace Hospital) of the Joint Logistic Support Force, Shijiazhuang, China

**Keywords:** appendicitis, single atrium and single ventricle, adult patient, anesthesia, low metabolic equivalent

## Abstract

Appendicitis is one of the common diseases, and appendectomy is one of the most commonly performed procedures. Single atrium and single ventricle are rare heart diseases, and very few patients survive to adulthood. We report a patient with single atrial and single ventricles undergoing appendectomy with transverse abdominis plane block and dexmedetomidine sedation anesthesia with smooth postoperative appendectomy.

## Introduction

Appendicitis is one of the most common surgical emergencies and appendectomy is one of the most commonly performed surgical procedures. The annual incidence of acute appendicitis (90–100)/100,000 people in developed countries are reported in the literature (1), about 50,000 appendectomies are performed annually in the UK and 300,000 in the USA (2). Chronic inflammatory lesions of the appendix, such as hyperplasia of the fibrous connective tissue of the duct wall, narrowing or occlusion of the duct lumen, distortion of the appendix, and adhesions to the surrounding tissues evolve into chronic appendicitis after acute appendicitis intervention or self-healing, which can reoccur or have multiple acute attacks.

## Case present

**FIGURE 1:**
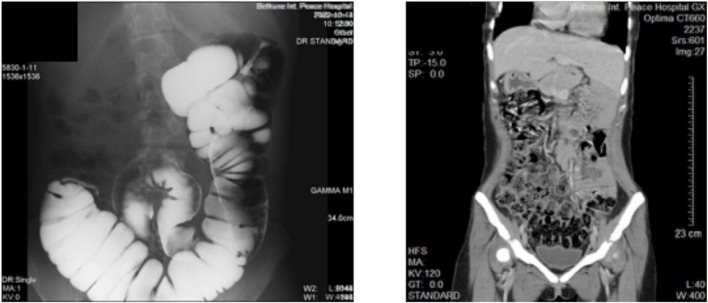
Imaging images of the patient’s abdomen. **(Left)** Barium examination. **(Right)** Full abdominal CT scan.

We now report a female patient, 20 years old, 44 Kg, with recurrent intermittent lower abdominal pain for more than 2 years, with exacerbation occurring 3 times, which improved significantly with interventions such as anti-inflammatory treatment when symptoms appeared. Half a month ago the symptoms reappeared and the patient was hospitalized for surgical treatment (Figure 1).

**FIGURE 2:**
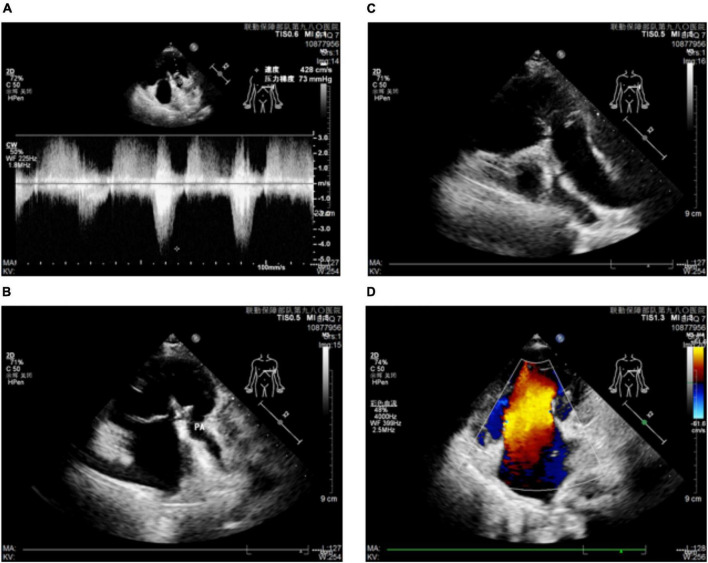
Ultrasound imaging of the patient’s heart. **(A,B)** Patient’s atrial ventricle and pulmonary artery imaging and pressure. **(C)** Images of the patient’s atrium ventricle and aorta. **(D)** Atrial ventricular flow imaging of the patient.

The patient had a history of cyanotic since birth and low activity tolerance. Due to the poor economic conditions, the family could not pay the bills in the early years. As she grew up, the best chance to cure her illness has been lost. Comprehensive consideration, she has not received surgical intervention. At the present, her metabolic equivalent (MET) was 4.0 [1 MET = 3.5 ml/(kg^.^min) of oxygen consumption] (3), and New York Heart Association Classification was Grade III. Electrocardiogram: sinus rhythm; P-wave peak, bimodal and widened; right-sided electrical axis, cis-clockwise transposition; RavR > 0.5 mV; right ventricular high voltage; ST-T abnormalities. Echocardiogram: (1). Her heart is located on the left side of the chest. Single ventricular end-diastolic volume was 63 ml, single ventricular end-systolic volume was 28 m1, and ejection fraction was 56%. The transverse diameter of the single atrium was about 50 mm, and the transverse diameter of the single ventricle was about 42 mm; (2). Muscular stenosis of the funnelus with an internal diameter of 1.6 mm, CDFI: flow rate of 376 cm/s, a pressure difference of 57 mmHg; Pulmonary valve thickened with a limited opening, annular diameter 10.2 mm, proximal aortic diameter 8.2 mm, distal aortic diameter 13.3 mm, CDFI: pulmonary artery opening velocity 327 cm/s, differential pressure 43 mmHg, moderate aortic regurgitation (3). Only one set of atrioventricular echoes could be detected, CDFI: flow rate of 436 cm/s, residence difference of 76 mmHg, moderate atrioventricular valve reflux (Figure 2).

Her oxygen saturation in room air (SpO2) was 72%, hemoglobin was 209 g/L, red blood cells were 8.84 × 10^∧12^/L, prothrombin time was 15.2 s, prothrombin activity was 65%, international normalized ratio (INR) was 1.35, total bilirubin was 60.8 umol/L, direct bilirubin was 9.2 umol/L, and other hematologic and biochemical tests were approximate. After a week of anti-inflammatory treatment to control the appendiceal inflammation, an open appendectomy under the left side’s transverse abdominal plane nerve block was proposed according to the patient’s wishes.

**FIGURE 3:**
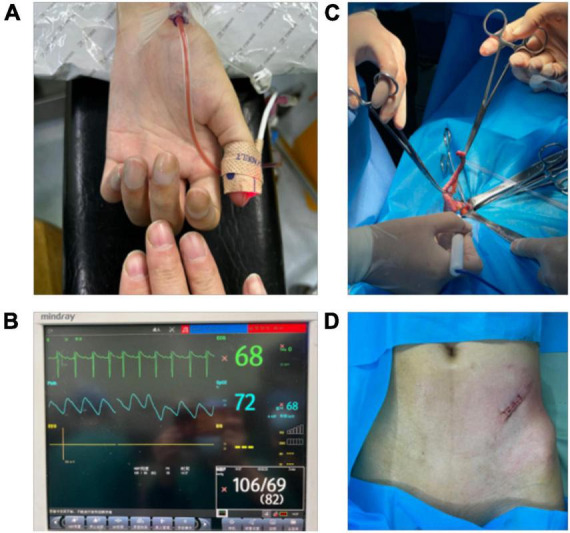
Images of patients during surgery. **(A)** Compared with the fingers of normal people, the finger color of the patient was purple. **(B)** Patient’s heart rate, blood pressure, and pulse oxygen saturation. **(C)** The moment when the patient’s appendix was removed. **(D)** Postoperative incision of the patient.

The patient was fasted for 8 hours and admitted to the operating room, where she was monitored for rhythm, heart rate, blood pressure, pulse oximetry, and arterial blood gas analysis followed by face mask oxygenation to improve oxygenation. The patient’s arterial blood gas analysis on admission showed: pH 7.363, PCO_2_ 36.4 mmHg, PO_2_ 39 mmHg, BEecf −5 mmol/L, HCO_3_ 20.7 mmol/L, TCO_2_ 22 mmol/L, sO_2_ 72%; after 10 min of oxygenation: pH 7.356, PCO_2_ 37.2 mmHg, PO_2_ 50 mmHg, BEecf −5 mmol/L, HCO_3_ 20.8 mmol/L, TCO_2_ 22 mmol/L, sO_2_ 84%; meanwhile, her venous blood gas analysis showed: pH 7.353, PCO_2_ 37.3 mmHg, PO_2_ 48 mmHg, BEecf −5 mmol/L, HCO_3_ 20.8 mmol/L, TCO_2_ 22 mmol/L, sO_2_ 81%. A left transversus abdominis plane block was performed with 20 ml of 0.125% ropivacaine under ultrasound guidance. After the patient’s sensation in the left lower abdomen was different from that in the right lower abdomen, the general surgeon used 0.5% lidocaine for local injection and intravenous pumping of dexmedetomidine 0.2 ug/kg/h. After the patient’s local abdominal wall sensation disappeared, the abdominal cavity was opened layer by layer by incision, and the blind end of the appendix was carefully searched for with long forceps and removed without any obvious adverse sensation during the operation. The operation lasted 45 min, 200 ml of Ringer’s fluid was infused, and bleeding was about 10 ml (Figure 3).

## Discussion

The patient’s multiple appendicitis episodes severely affected her daily life and became another significant burden for her co-morbid heart disease. Although theoretically, this appendicitis could still be relieved with medications and other interventions, the patient’s desire for surgery was strong and we respected the patient’s wishes to finalize our treatment decisions with a patient-centered approach (4). Recurrence after conservative treatment of acute appendicitis is also a concern for both physicians and patients. The APPAC trial with a 72.7% success rate of conservative treatment was reported to have a recurrence rate of 27.3, 34.0, 35.2, 37.1, and 39.1% at 1–5 years after 5 years of follow-up (5). The recurrence rate in real-world studies may be lower. Data from the National Health Insurance in Taiwan, China showed that 15.1% of nearly 240,000 first-time hospitalizations for acute appendicitis received non-surgical treatment, with an average follow-up of 6.5 years and 7.1% of recurrences (6). 1.5% of nearly 240,000 cases of uncomplicated acute appendicitis in 11 years were treated non-operatively, 5.9% failed, and 4.4% recurred (median follow-up > 7 years), with a lower recurrence rate in women than in men, California, USA (7). Darwazeh et al. (8) systematically reviewed 21 non-operative treatments for appendiceal abscess or cellulitis with a mean follow-up of 45.9 months and a recurrence rate of 0∼24% with a mean of 12.4%. Surgery should be recommended for cases with multiple (≥ 2) recurrences, subacute or chronic appendicitis that appears to be successfully treated conservatively but still has abdominal pain, or the occurrence of postoperative stump inflammation. During this procedure, the surgeon visually observes inflammation in the appendix. Studies have shown that the surgeon’s intraoperative visual determination of the type of appendiceal pathology is approximately 80% accurate (9). Postoperative pathology also confirmed the patient’s diagnosis of chronic appendicitis.

In patients with a common atrium and single ventricle, arterial and venous blood are mixed in the common cardiac chamber and decreased pulmonary blood flow is due to infundibular stenosis. Patients with single atrium and single ventricle heart disease are rare and account for 1∼2% of all congenital heart malformations. Without corrective surgery, it will die in the neonatal period or infancy, and survival into adulthood is unusual (10). Patients with single atrium and single ventricle hearts require surgery in infancy or childhood to ensure balanced pulmonary and systemic blood flow (11). Due to the disturbance of the homeostasis of their hemodynamic state, few patients can live an almost normal life without any surgical intervention. Only about 10 cases worldwide have been reported for patients with single ventricle surviving to late adulthood, one of them up to 62 years of age and the others up to 50 years of age (12). Survival to adulthood in patients with a single ventricle combined with a single atrium is even rarer, with only a very few cases reported so far (13, 14). There have been few reported cases of patients successfully surviving into adulthood with concurrent surgical treatment. In addition to a primigravida with a single atrium and single ventricle (15), and a patient with anomalous hepatic venous drainage and azygos continuation of inferior vena cava (16).

It is a miracle that the patient survived to adulthood, which may be related to the tolerable pressure of the pulmonary artery trans-valve. The patient also paid attention to the prevention of infectious diseases such as colds and the amount of exercise she took. According to relevant calculations, the patient’s metabolic equivalent is currently assessed at 4 points, and she can almost perform simple daily life independently. Interventions in such patients focus on maintaining the patient’s cardiac and pulmonary circulatory homeostasis and reducing disturbances to cardiac function that she has tolerated for many years. Considering that the patient had chronic appendicitis and that the local inflammation and adhesions of the appendix were not too severe, the general surgeon, cardiac surgeon, and anesthesiologist unanimously decided to use regional block anesthesia instead of general anesthesia to maintain the balance of cardiac and pulmonary function in this patient. A patient with Marfan syndrome receiving transversus abdominis plane blocks with analgesic results has been reported (17) and administration of dexmedetomidine might reduce the adverse effects of general anesthesia in infants with congenital heart disease undergoing surgery and extracorporeal circulation (18). The surgical showed that ultrasound-guided transversus abdominis plane block and dexmedetomidine had a less hemodynamic impact on this patient, and reduced the patient’s tension, anxiety, and discomfort, creating conditions for a successful surgery. The patient had almost no adverse effects during the operation, and the appendectomy was completed successfully.

## Data availability statement

The original contributions presented in this study are included in this article/supplementary material, further inquiries can be directed to the corresponding authors.

## Ethics statement

Written informed consent was obtained from the individual(s) for the publication of any potentially identifiable images or data included in this article.

## Author contributions

YW and BZ took the responsibility of communicating with the patient’s family and obtaining authorization for this manuscript. YW was responsible for drafting the manuscript. WL revised the manuscript. ZZ, ZC, and JC were responsible for literature searches and final proofreading. All authors contributed to the article and approved the submitted version.
